# An Aboriginal Community-Controlled Health Organization model of service delivery: qualitative process evaluation of the Tulku wan Wininn mobile clinic

**DOI:** 10.1186/s12939-022-01768-4

**Published:** 2022-11-16

**Authors:** H. Beks, F. Mitchell, J.A. Charles, K.P. McNamara, V.L. Versace

**Affiliations:** 1 Deakin Rural Health, School of Medicine, Warrnambool, Victoria Australia; 2 First Peoples Health Unit, Health Group, Griffith University, Queensland, Australia

**Keywords:** Global health, Health services, Indigenous health, Mobile health clinics, Primary health care, Health Services, Indigenous, Aboriginal, Qualitative, Rural health services

## Abstract

**Background:**

Mobile clinics have been implemented in diverse clinical and geographical settings to provide proximal health care for specific populations. Primary health care mobile clinics have been implemented widely for Indigenous populations, with a paucity of research evaluations around service delivery models internationally. To redress factors impeding service accessibility for Aboriginal and Torres Strait Islander Peoples, Budja Budja Aboriginal Cooperative (Aboriginal Community Controlled Health Organisation located in a small rural town in Victoria, Australia), developed and implemented the Tulku wan Wininn primary health mobile clinic.

**Methods:**

A qualitative process evaluation methodology was used to explore contextual factors mediating the implementation of the mobile clinic, including the acceptability of the service to health service personnel, external key informants, and Aboriginal and/or Torres Strait Islander clients. A synthesis of international ethical guidelines, (Consolidated Criteria for strengthening reporting of health research involving Indigenous peoples (CONSIDER statement), was prospectively applied to shape the study design and research process. Semi-structured interviews were conducted with participants. Data collection occurred from July 2019 to October 2021. Inductive thematic data analysis was undertaken concurrently with data collection.

**Results:**

Data was collected from 19 participants which included 12 health service personnel and key informants, and 7 Aboriginal clients. In total, data from 22 interviews were included as interviews with three clients were undertaken twice. Four themes were developed: considerations for early implementation, maintaining face-to-face services during COVID-19, acceptability as a model of service delivery, and maintaining the mobile clinic as a service delivery model.

**Conclusion:**

Evidence supporting the acceptability of a primary health care mobile clinic for Aboriginal Peoples residing in rural Victoria is provided. Despite the experience of early implementation challenges and adaptations, the mobile clinic addressed known transport and cultural barriers to accessing primary health care services. In the context of COVID-19 lockdowns, the mobile clinic was valued for the provision of face-to-face care for Aboriginal clients. Key issues for maintaining the mobile clinic include health workforce and funding. Findings are of value to other organizations seeking to implement a primary health mobile clinic service delivery model to redress barriers to accessibility experienced by the communities they serve.

**Supplementary Information:**

The online version contains supplementary material available at 10.1186/s12939-022-01768-4.

## Background

Mobile clinics in the form of vans and buses, have been implemented in diverse geographical and clinical settings to provide proximal health care for specific populations [1–5]. Examples include mobile specialist screening services (e.g., for retinal screening [6], mammography [3]), treatment services (e.g., dialysis for end-stage kidney disease) [7], and mobile clinics for populations affected by natural disasters [2]. Although global research supporting mobile clinics is sparse, evidence from the United States supports mobile clinics as a model of service delivery which improves access to health services, particularly for otherwise vulnerable communities (e.g., homeless persons, rural populations, minority groups) and populations that are hard to reach [4, 8].

Primary health care mobile clinics for Indigenous Peoples (a term reserved for the global context) have also been widely implemented in high-income colonized countries such as Australia, Canada, and the United States [5, 9]. A review examining primary health care mobile clinics implemented for Indigenous Peoples in these countries identified a paucity of evaluation evidence supporting these clinics despite widespread implementation [9]. Specifically, key gaps included a lack of qualitative evidence around the acceptability of mobile clinics from the perspectives of Indigenous Peoples and the cost-effectiveness of mobile clinics when compared to other service delivery models (e.g., a fixed clinic) [9]. Despite these evidence gaps, a shared theme of these primary health mobile clinics is to provide culturally appropriate and holistic primary health care where Indigenous Peoples reside (e.g., on Country, on Missions or Reserves) [9]. Further, mobile clinics have been implemented to target health inequities experienced by Indigenous Peoples (e.g., high prevalence of morbidity and barriers to health service accessibility such as racism and transport) [10, 11]. Examining whether mobile clinics are achieving these objectives by undertaking scientifically and ethically rigorous evaluations which engage with the perspectives of Indigenous Peoples, is necessary [9].

Establishing partnerships with Indigenous health organizations and communities is key to this [12–18]. Strong support is offered for the role of community-based and governed health services to implement service delivery models, which meet the health care needs of Indigenous Peoples [19]. This is also congruent with the international principles of self-determination and Indigenous rights [20, 21]. To meet this need in Australia, there are over 140 Aboriginal Community Controlled Health Organizations (ACCHOs) governed by Aboriginal and Torres Strait Islander communities which are geographically located within each respective community [22]. These health organizations are generally valued by Aboriginal and Torres Strait Islander Peoples for the provision of culturally safe and comprehensive care [23, 24].

As an ACCHO located in a small rural town (Halls Gap, Victoria, Australia) on Djab Wurrung and Jardwadjali Country which serves a large geographical area (10,000 square kilometers), Budja Budja Aboriginal Cooperative (BBAC) sought to develop solutions to improving the accessibility of primary health care services for Aboriginal and Torres Strait Islander Peoples in their service region through a community consultation process [25]. Transport barriers (e.g., lack of access to own or shared private transport, no public transport, travel cost and time) were frequently cited as an issue during consultation. The concept of a mobile clinic model of service delivery was then developed. However, scant evidence regarding the implementation of primary mobile clinics within Aboriginal and Torres Strait Islander communities was available to guide the development of the service model.

An academic partner was sought by BBAC to develop evidence around the emerging mobile clinic service delivery model. Deakin Rural Health (DRH), a University Department of Rural Health (UDRH) located in Warrnambool (Victoria, Australia) established under the Australian Government Rural Health Multi-Disciplinary Training (RHMT) funding [26], was approached in December 2017. Over two years, a partnership was established between BBAC and DRH [27]. This led to the co-design and development of a service plan for the mobile clinic led by BBAC and a research evaluation plan (internal documents). In July 2019, the Tulku Wan Wininn mobile clinic (meaning ‘Health to You’ in local Djab Wurrung language) was implemented [27]. The Tulku wan Wininn mobile clinic provides general practitioner (GP), allied health (including optometry and audiology services), and social and emotional wellbeing services close to where Aboriginal and/or Torres Strait Islander Peoples reside. Services are also delivered through schools and at residential corrective service facilities. Using qualitative inquiry to explore factors mediating implementation including the acceptability of the service, was identified as key to developing the evidence base around this model of service delivery by addressing gaps in the literature [9]. The Consolidated criteria for Reporting Qualitative studies (COREQ) was used to guide the reporting of this study (Supplementary File 1) [28].

### Aim of the study

To undertake a qualitative process evaluation exploring factors mediating the first two years of implementation, including the acceptability of the service from the perspectives of health service personnel, external key informants, and Aboriginal and/or Torres Strait Islander clients.

### Ethics approval

Ethical approval was obtained through Deakin University Human Research Ethics Committee (DUHREC protocol number 2019 − 432) and through the BBAC Board evidenced through letters of support which were submitted with the institutional ethics application. In Victoria, there is currently no designated Aboriginal and Torres Strait Islander Human Research Ethics Committee to review research studies involving Aboriginal and/or Torres Strait Islander Peoples. The Victorian Aboriginal Community Controlled Health Organization (the peak representative body for ACCHOs in Victoria, Australia) is developing guidance around this. 

## Methods

### Study design

A process evaluation methodology was used to shape the qualitative study design. A similar approach has been applied by other research undertaken in the primary care setting [29]. Process evaluations involve identifying factors mediating implementation and examining whether the intervention was implemented as planned [30, 31]. The Consolidated Criteria for strengthening reporting of health research involving Indigenous Peoples (CONSIDER statement) which is a synthesis of international ethical guidelines, was used to guide the study design and research process using a community-based approach [32]. The CONSIDER statement has also been used as a reporting checklist (Supplementary File 2) – an approach used in another community-based study undertaken in the rural ACCHO context [32, 33]. Inherent to the research evaluation process is establishing genuine partnerships with Indigenous Peoples embedded in an understanding of Indigenous self-determination, ownership, governance, and data sovereignty [34–36]. Qualitative data was collected during the first two years of implementation (July 2019 to June 2021) and during a follow up period (July 2021-October 2021).

### Setting and intervention

The Tulku wan Wininn mobile clinic was implemented in July 2019 for Aboriginal and Torres Strait Islander Peoples residing in the BBAC service region (Northern Grampians and Ararat regions of Victoria, Australia). The BBAC fixed clinic is located in a small rural town (Modified Monash 5) as defined by the Modified Monash Model which is a geographical classification used by the Australian Government Department of Health consisting of seven categories [37]. Outside of metropolitan (MM1) and regional areas (MM2), small rural towns (MM5) hold the highest proportion of the Australian population when compared to the remaining categories (MM3 - large rural towns, MM4 – medium rural towns, MM6 – remote communities, MM7 – very remote communities) [38]. As a clinical van fitted with medical equipment and a consultation suite for patients, the mobile clinic facilitates the provision of general practitioner, nursing, and allied health services. Services are provided in multiple locations within the BBAC service region, including schools, residential corrective services facilities, and public spaces (e.g., parks).

### Participant sample and recruitment

Health service personnel, external key informants, and Aboriginal and/or Torres Strait Islander clients/family members of clients of the mobile clinic service, were purposively sampled [31]. Health service personnel included health professionals (including locum health professionals). non-clinical staff and managerial staff working at BBAC who were involved in the development and/or implementation of the mobile clinic service. Key informants were persons external to BBAC from government and health organizations, who were key to the development of the mobile clinic model of service delivery. Clients included Aboriginal and/or Torres Strait Islander adult clients of BBAC (persons aged 18 years and above) and/or adult family members of clients, who had accessed services through the mobile clinic.

Health service personnel and external key informants were approached directly by the researchers using telephone or email and were invited to participate in a semi-structured interview. Those interested in participating were provided with a Plain Language Statement (PLS) detailing the purpose of the study and nature of involvement, and a consent form. A mutually convenient time was arranged between the participant and the researchers to meet remotely using a videoconferencing platform. Face-to-face interviews were not possible due to lockdowns in the context of the COVID-19 pandemic. Prior to commencing the interview, written informed consent was obtained.

To recruit Aboriginal and/or Torres Strait Islander clients/family members of clients, a letter with details about the study was sent from BBAC to all clients who had accessed the Tulku wan Wininn mobile clinic. This letter offered the opportunity to participate in an interview with researchers. Clients who expressed interest were contacted by researchers once they had granted permission to BBAC for their name and phone number to be provided to the external researchers. A PLS was provided with more information about the research. A mutually convenient time was arranged to meet remotely using the telephone or videoconferencing (depending on the preferences of participants). Written informed consent was provided by participants prior to commencing the interview. A $50 voucher (AUD) was provided to participants to reimburse them for their time.

### Data Collection

Semi-structured interviews were undertaken remotely (videoconferencing or telephone) with health service personnel, external key informants, and clients. Two interview guides were used (Supplementary File 3). Interviews were undertaken by two female researchers and two male researchers external to BBAC at the time and who had previous experience of undertaking research (HB, FM, JAC, and VLV). Two of the researchers were Aboriginal (FM and JAC) and guided the cultural conduct of interviews, particularly those undertaken with clients. Researchers had no relationships with clients before the research but were known to some health service personnel and external key informants due to the community-based nature of the study. Repeat interviews were undertaken with some clients as part of the process of developing rapport. Researchers engaged in a debrief following interviews to discuss the data and emerging concepts, as well as the role of researchers on the nature of dialogue (reflexivity) – particularly for interviews where the participant was known to the researcher [39]. Interviews were audio-recorded and transcribed verbatim by the researchers involved in data collection. A copy of the written transcripts was provided to participants which also allowed an opportunity for participants to provide any feedback on the content of the interviews.

### Data analysis

Data analysis was an iterative process undertaken concurrently with data collection due to the longitudinal nature of the study period. This also allowed emerging concepts from analysis to be explored further in subsequent interviews. Data from semi-structured interviews were analyzed thematically using an inductive process by a team of researchers (HB, FM, JAC) [40, 41]. This involved an independent initial reading of the transcripts by at least two researchers and the use of open descriptive coding as a first cycling method [42]. Findings and emerging concepts were discussed between researchers. Focused coding was then used as a second cycling method to develop key concepts [42]. A thematic framework was developed through this process. Themes were then reviewed by two other researchers with experience in qualitative research (KPM, VLV). To support data analysis, QSR NVivo for Windows, version 12 (QSR International Pty Ltd., Melbourne, Vic, AU) was used.

### Rigor of qualitative methods

Strategies to improve the scientific and ethical rigor of qualitative methods were guided by Lincoln and Guba’s (1985) criteria of trustworthiness in qualitative research (i.e. credibility, transferability, dependability, and confirmability) [43] and the principles of the CONSIDER statement (Supplementary File 2) [32]. A prolonged period of engagement with participants was one strategy to improve the credibility of findings which enabled validating emerging concepts from concurrent data analysis with participants through member checking. To improve dependability and confirmability, researchers engaged in frequent informal debriefs and reflexive practice to discuss emerging concepts. An established partnership with BBAC guided the ethical conduct of the study in addition to the expertise and cultural knowledge of Aboriginal researchers involved in the study design, data collection, and analysis.

## Results

From July 2019 to November 2021, 12 health service personnel and external key informants, and 7 Aboriginal clients participated in a qualitative interview. One client had not directly utilized the mobile clinic but was a carer for a client who had used the mobile clinic. No participants identified as Torres Strait Islander. A total of 22 interviews were undertaken (interviews with three clients were repeated as part of developing rapport) (Table 1). Interviews ranged from five minutes to fifty minutes. The three interviews that had a duration of five minutes were undertaken with three clients and were repeated at two different time intervals to examine the experience of clients accessing the service over time.

**Table 1:** Interview participant demographics

Participant group	No. of participants (male: female: other)	No. of interviews
Health service personnel	10 (1: 9: 0)	10
External key informants	2 (1: 1: 0)	2
Aboriginal clients	7 (3: 3: 1)	10*
Total	19	22

*Interviews were repeated with three clients

Four themes were developed: considerations for early implementation, maintaining face-to-face services during COVID-19, acceptability as a model of service delivery, and maintaining the mobile clinic as a service delivery model.

### Considerations for early implementation

Three considerations were identified during early implementation of the Tulku wan Wininn mobile clinic. These were: early implementation challenges, importance of awareness raising at a community level, and the need to adapt the model of service delivery.

### Early implementation challenges

Early implementation challenges identified by health service personnel included mechanical issues with the mobile clinic which required the van to be returned to the manufacturer for repairs. Other technical issues included running out of electricity due to insufficient battery storage and having difficulty setting up the satellite so health professionals could access client medical records. From an administrative viewpoint, logistical issues were also experienced such as obtaining the necessary council permits to park the mobile clinic in a public space that was accessible to clients, and ensuring that the appropriate utilities (e.g., electricity supply), and amenities (e.g., toilets) were nearby. Having multiple locations to service in one day by the mobile clinic was also identified as challenging as medical equipment would have to be stored for transit and then set up, requiring an additional demand on time. Other administrative issues included aligning appointment times with school hours (for school-based clinics). Operational issues were also experienced when setting up the mobile clinic’s physical space for patient consultations. This included ensuring adequate sound proofing for audiology consultations and identifying the most appropriate way to manage potential risks, such as client aggression, within the confines of the mobile clinic. Sourcing the necessary medical equipment to fit the physical space of the mobile clinic (e.g., electrocardiograph machine, defibrillator), was also required.

### Importance of awareness raising at a community level

To navigate some of these challenges, health service personnel and key informants identified the importance of raising awareness around the model of service delivery at a community level, which included with other health services and organizations in the region. The role of the Mobile Clinic Coordinator, a position established to project manage implementation, was identified as a strategic engagement role instrumental to this process. Activities undertaken by the Mobile Clinic Coordinator during early implementation included contacting schools in the region through Aboriginal Education Officers, liaising with other health services, including with Aboriginal Health Workers, and using media and flyers to raise awareness of the mobile clinic with Aboriginal and Torres Strait Islander Peoples in the BBAC service region. This was largely understood to be a relational process requiring time and the development of respectful partnership with other stakeholders in the region.

… the first twelve months has actually been raising the awareness of the van which is typical of what you do for a new service. You raise the awareness, and you raise the community acceptance, and you get the word of mouth through the community. – Health Service Personnel

### Need to adapt the model of service delivery

Adaptations were required to address contextual issues, both internal and external to BBAC. Internal contextual issues impacting the early implementation period included changes in BBAC staffing. The commencement of a new Mobile Clinic Coordinator saw a change in the position from establishing the service model to building the client base of the mobile clinic using a more targeted approach which involved following up with other organizations (e.g., schools). The most significant external contextual issue with the potential to disrupt the early implementation of the mobile clinic services, was the COVID-19 pandemic and associated lockdowns by the Victorian Government in regional Victoria which occurred from March 2020 to September 2021. It was observed by health service personnel that COVID-19 slowed the momentum of expanding the reach of the mobile clinic to Aboriginal and/or Torres Strait Islander Peoples in the service region as it led to the cancellation of planned events and of outreach allied health services supplied by external organizations. Although navigating COVID-19 presented challenges for health service personnel, the mobile clinic model of service delivery was important in maintaining face-to-face services to clients during lockdowns.

### Maintaining face-to-face services during COVID-19

During the first COVID-19 lockdown in regional Victoria (March 2020), health service personnel observed that the momentum of consultation uptake by Aboriginal and/or Torres Strait Islander clients slowed. This was attributed to the need to implement physical distancing measures. Attendance increased as adaptations were made to the mobile clinic service delivery model to meet the reported needs of clients who were feeling isolated during this period. At this time, the Australian Government had also established funding for telehealth services in primary care settings. This led to an integration of telehealth services (including both telephone and videoconferencing services) and the face-to-face services of the mobile clinic to deliver a hybrid model of service delivery during lockdown periods. Health service personnel reflected that maintaining face-to-face service delivery to clients was particularly important during COVID-19 as BBAC were unable to provide transport services to the fixed clinic due to lockdown restrictions. Further, the mobile clinic was also used to deliver the annual influenza vaccines to clients which was identified as another benefit.

…it [mobile clinic appointments] were starting to pick up and then of course COVID hit so we were a bit limited but we’re actually using the bus [mobile clinic] a bit more because we can’t provide transport anymore, we’re using the mobile clinic one to two times a week. We’ve had a few mobile flu shot clinics we’ve taken to [name of suburb] and [name of suburb]. – Health service personnel

The need to constantly adapt to COVID-19 lockdown restrictions to maintain face-to-face service delivery through the mobile clinic was discussed at length. This included the wearing of personal protective equipment and implementation of infection control procedures. Balancing this with the needs of clients (e.g., need for an face-to-face health assessment) was an iterative process requiring constant monitoring. From the perspectives of health service personnel, clients had found the COVID-19 lockdowns challenging, particularly those who were experiencing concurrent mental health issues. In this way, the mobile clinic was a valuable resource used to check in with clients.

…we’ve had to become very creative in how we support our community members. – Health Service Personnel

### Acceptability as a model of service delivery

From the perspectives of Aboriginal clients, health service personnel, and external key informants, the mobile clinic was an acceptable model of service delivery. A key strength was bringing primary health care services to where clients geographically reside. It was widely identified that the mobile clinic addressed known barriers to accessing the BBAC fixed clinic in Halls Gap which included not having access to transport (and not being able to afford transport or able to drive), having family commitments, homelessness, living in a supervised facility following release from a correctional facility, having a disability, and having work commitments.

Aboriginal clients appreciated the convenience of the mobile clinic in providing GP and allied health services. However, some clients conveyed a preference for attending the BBAC fixed clinic in Halls Gap as they found this had more social benefits such as generally catching up with other clients and staff. Despite this preference, clients found the mobile clinic to be of particular importance during the COVID-19 lockdowns to receive face-to-face care, particularly when BBAC transport to the fixed clinic was not available. Without the mobile clinic, some clients reflected that it would have been difficult for them to see a GP, both during COVID-19 (due to transport issues) and beforehand (due to not feeling comfortable in attending another medical clinic).

I mean I like the social side of going out to Halls Gap, to Budja, but with this COVID thing, the bus [mobile clinic] has been a blessing, otherwise we wouldn’t have been able to see the doctor. We couldn’t have done without it during this COVID thing. – Client

…it’s exactly like a medical [clinic], it’s more private. I like it more because it’s private and I don’t get anxiety and all of that sort of thing with the bus [mobile clinic], but I don’t think, I know I am not very good at going to medical clinics. – Client

Key to the mobile clinic being acceptable from the perspectives of clients was the care provided by health service personnel delivering the mobile clinic service due to the buildup of trust and relationships with individual clients over time, particularly during COVID-19. Aspects of care that were appreciated by clients included individual follow up in the form of telephone calls and visits, coordination of food hampers, facilitation of social events using the mobile clinic, and generally taking additional measures to support clients. Rapport and care provided by health service personnel coordinating the mobile clinic was fundamental to ensuring the service met client needs.

I like it when they [BBAC] come to [name of suburb], with their bus [mobile clinic] and all that. If I have to see [name of GP] for anything like that. If I need a helping hand… I get anything and I don’t understand it, I just ask them. – Client

From the perspectives of health service personnel, the mobile clinic was also particularly useful for older people, clients with chronic disease, multi-morbidity (including mobility issues) or complex needs (e.g., homelessness), families (particularly those with multiple children), and for providing screening services in schools (e.g., audiology and optometry).

And what I think it [mobile clinic] is really good for, is families with young children. That they don’t have to all pile in the car and be able to all get out here [BBAC fixed clinic]. The idea that they, we [BBAC] can come to them and do their health assessments with their kids just makes life a whole lot easier for them. – Health Service Personnel

### Maintaining the mobile clinic as a service delivery model

Participants reflected on key considerations for maintaining the mobile clinic as a service delivery model. These related to the health workforce, utilization of services, funding, and ensuring the service met the needs of clients. Health service personnel and clients identified that having accessible general practitioner services was imperative for maintaining the mobile clinic model of service delivery. This was challenging during implementation given the loss of the BBAC permanent GP and subsequent reliance on the locum GP workforce. A preference for a GP who was known to clients and who was aware of their medical history, was voiced. Another key issue was that GP-generated revenue was the main source of funding from the MBS. .

Implementing targeted strategies to continue expanding the reach of the mobile clinic to Aboriginal and/or Torres Strait Islander Peoples in the BBAC service region was also identified as important to maintaining the mobile clinic from the perspectives of health service personnel. Without the utilization and support of Aboriginal and Torres Strait Islander Peoples, it was identified that the service would not be justified or adopted by other health organizations. Examples of targeted strategies to increase the mobile clinic client base including focusing on geographical areas where Aboriginal and Torres Strait Islander Peoples are known to reside, keeping community members informed of mobile clinic dates and locations through various media, and providing services in partnership with other organizations (e.g., schools, residential facilities).

General feedback provided by clients to improve the service that had implications for maintaining the mobile clinic, included making modifications to the physical space to ensure it was more accessible for clients requiring mobility aids (e.g., wheelchair ramp), expanding the delivering of clinical services offered (e.g., dentist, routine pathology testing), and improving the continuity of care when using locum GPs (e.g., familiarity with medication history). Both health service personnel and clients were supportive of the ongoing implementation of the mobile clinic in their community. There was a general understanding that the priorities of the mobile clinic were guided by the needs of clients utilizing the service.

…what’s the good of buying something like that [mobile clinic], just sitting and watching it fade away. We’ve got to utilize it otherwise they won’t replace it. But like I say, it’s a great asset to the community and it’s open to everybody… it’s for everybody who feels like they need to see the doctor. - Client

## Discussion

This study provides a qualitative contribution to the evidence around primary health care mobile clinics [1] – specifically, a mobile clinic implemented for an Aboriginal and Torres Strait Islander community in rural Victoria [9]. Findings from the process evaluation highlight considerations for early implementation, including early implementation challenges and administrative issues. Support for the use of mobile clinics in maintaining face-to-face services in the context of the COVID-19 pandemic is also provided. Overall, the Tulku wan Wininn mobile clinic is an acceptable model of service delivery from the perspectives of health service personnel and Aboriginal clients. Key considerations for maintaining the mobile clinic include having sufficient health workforce availability, funding, expansion into an array of various health and medical services and ensuring that the mobile clinic continues to meet the needs of clients. Findings are of value to other health organizations seeking to implement of primary health mobile clinic model of service delivery.

Early implementation challenges identified in this study expand on the unique issues of mobile clinics already documented in the literature. These include logistical challenges, in addition to space and structural constraints (e.g., reliance on generators, internet accessibility) [1]. For mobile clinics to be successfully implemented, it is necessary for these issues to be addressed which will vary across different settings [1]. The need to address contextual variations is typical when implementing new or existing interventions in other settings [44]. The appointment of a designated mobile clinic coordinator was imperative to guiding the early implementation of the Tulku wan Wininn mobile clinic and managing operational issues (e.g., technology, batteries, mechanical failures) attributed to the setting. As a result, adaptions were made as the model of service delivery was refined. If a similar model of service delivery were implemented in another community or setting, the position of mobile clinic coordinator would be of value to oversee early implementation and identify contextual issues to guide the adaptation process [44, 45]. Further, this is an important mechanism to ensuring the mobile clinic meets the needs of clients.

Support for the implementation of mobile clinics to maintain the delivery of health care to otherwise hard to reach communities (e.g., homeless persons) during the COVID-19 pandemic also gained traction in other settings, particularly within the United States [8, 46, 47]. Key functions of these clinics included providing telehealth services, community education about COVID-19, diverting people from hospitals, and distributing emergency food [46]. In Australia, mobile clinics were also used to roll out the COVID-19 and influenza vaccinations and other primary health care services to communities requiring additional support [48]. This was particularly important in Victoria where extended lockdowns were implemented by the Victorian government which impeded the movement of the population and limited the activities people could engage in. To our knowledge, this is the first qualitative evaluation examining the implementation of a primary health care mobile clinic during the COVID-19 pandemic which captures challenges encountered and adaptations made.

The maintenance of face-to-face services and provision of proximal primary health care to where clients reside particularly during COVID-19, were key findings around the acceptability of the mobile clinic. Findings expand on qualitative research evaluating a mobile dialysis clinic for Aboriginal and Torres Strait Islander Peoples implemented in South Australia [7] which found the clinic to be an acceptable model of service delivery and culturally appropriate. Further qualitative research is required exploring the acceptability of mobile clinic models of service delivery for Indigenous Peoples across a number of countries globally and across a range of different clinical settings [9]. The availability of the mobile clinic during COVID-19 to maintain face-to-face services was timely. It is likely that it would have been difficult for clients to access primary health care services through other means during this period, particularly as BBAC were unable to provide transport services to the fixed clinic due to COVID-19 restrictions.

Although this qualitative process evaluation study explored factors mediating the implementation of the mobile clinic, issues pertinent to maintaining the service were identified by participants. This included attracting sufficient funding and maintaining a GP workforce. In the evidence around mobile clinics, funding has been cited as a challenge due to the reliance on multiple funding sources, including grants from philanthropic organizations [1, 9]. The maldistribution of the health workforce is an established issue in rural and remote geographical regions [49] and is evident in the current study by the challenges encountered to maintaining a GP.

Subsequent research should expand on these issues and consider the sustainability of mobile clinics in rural contexts and in Indigenous health organizations. In general, integrating evaluation into the design and implementation of new interventions, particularly within the Aboriginal and Torres Strait Islander health context, is imperative to meeting these knowledge gaps [44, 45]. At BBAC, funding for an evaluation officer has been obtained to provide continuity in evaluating new models of health service delivery at the ACCHO in partnership with DRH – the external researchers in this study.

## Limitations

This study provides a qualitative process evaluation engaging with the experiences of a sample of health service personnel, external key informants, and Aboriginal clients in developing, implementing, and accessing a primary health care mobile clinic model of service delivery at a single rural ACCHO. Data was collected during COVID-19 when lockdowns were in place in Victoria, Australia. Due to this, interviews were unable to be conducted in person. The Indigenous research method of yarning was originally going to be applied to obtain feedback from clients. This was not possible as the yarning process requires face-to-face communication and interaction. Engaging in this process over either the telephone or videoconference is not culturally appropriate. Further, no clients identifying as Torres Strait Islander participated. Future research should undertake cost-effectiveness studies of mobile clinics as a service delivery model for Indigenous People and examine the impact of this service delivery model on health outcomes.

## Conclusion

Evidence supporting the acceptability of the Tulku wan Wininn primary health care mobile clinic model of service delivery for Aboriginal Peoples residing in rural Victoria, Australia, is provided. Despite the early implementation challenges and adaptations, the Tulku wan Wininn mobile clinic addressed known transport and cultural barriers to accessing primary health care services. In the context of COVID-19 lockdowns, the mobile clinic was valued for maintaining the provision of face-to-face care for clients. Key issues for maintaining the mobile clinic include health workforce and funding. Findings are of value to other organizations seeking to implement a primary health mobile clinic service delivery model to redress barriers to accessibility experienced by the communities they serve. Further, this study provides a contribution to the growing international evidence base around mobile clinic models of service delivery.

## Electronic supplementary material

Supplementary Material 1. Consolidated criteria for reporting qualitative studies (COREQ): 32-item checklist. 

Supplementary Material 2. Application of the CONSIDER Statement. 

Supplementary Material 3. Interview Guides.

## Data Availability

The data generated and analyzed in this study are not publicly available as participants consented to their data being used only for the purposed described in this study.

## Abbreviations

ACCHO: Aboriginal Community Controlled Health Organisation
BBAC: Budja Budja Aboriginal Cooperative
DRH: Deakin Rural Health
GP: General Practitioner
MBS: Medicare Benefits Schedule
RHMT: Rural Health Multidisciplinary Training
