# Supplementary material for: An aboriginal community-controlled health organization model of service delivery: qualitative process evaluation of the Tulku wan Wininn mobile clinic
Source: Int J Equity Health. 2022 Nov 16;21:163. doi: 10.1186/s12939-022-01768-4 (PMC9667861; doi:10.1186/s12939-022-01768-4)
Supplement: Supplementary file 1 — Supplementary Material 1. Consolidated criteria for reporting qualitative studies (COREQ): 32-item checklist. [file 12939_2022_1768_MOESM1_ESM.docx]

**Supplementary File 1. Consolidated criteria for reporting qualitative studies (COREQ): 32-item checklist***

| **No** | **Item** | **Guide questions/description** |
| --- | --- | --- |
| **Domain 1: Research team and reflexivity** |  |  |
| Personal Characteristics |  |  |
| 1. | Interviewer/facilitator | Which author/s conducted the interview or focus group? **Page 9** |
| 2. | Credentials | What were the researcher's credentials? **Beks H (RN, MPH), Mitchell F (GradDipIndRes), Charles JA (PhD), Mc Namara KP (PhD), Versace VL (PhD)** |
| 3. | Occupation | What was their occupation at the time of the study? **Page 9** |
| 4. | Gender | Was the researcher male or female? **Page 9** |
| 5. | Experience and training | What experience or training did the researcher have? **Page 9** |
| Relationship with participants |  |  |
| 6. | Relationship established | Was a relationship established prior to study commencement? **Page 9 and 10** |
| 7. | Participant knowledge of the interviewer | What did the participants know about the researcher? e*.g. personal goals, reasons for doing the research* **Page 9 and 10** |
| 8. | Interviewer characteristics | What characteristics were reported about the interviewer/facilitator? e.g. *Bias, assumptions, reasons and interests in the research topic* **Page 9 and 10** |
| **Domain 2: study design** |  |  |
| Theoretical framework |  |  |
| 9. | Methodological orientation and Theory | What methodological orientation was stated to underpin the study? *e.g. grounded theory, discourse analysis, ethnography, phenomenology, content analysis* **Page 7** |
| Participant selection |  |  |
| 10. | Sampling | How were participants selected? *e.g. purposive, convenience, consecutive, snowball* **Page 8 and 9** |
| 11. | Method of approach | How were participants approached? e*.g. face-to-face, telephone, mail, email* **Page 8 and 9** |
| 12. | Sample size | How many participants were in the study? **Page 11** |
| 13. | Non-participation | How many people refused to participate or dropped out? Reasons? **Not applicable** |
| Setting |  |  |
| 14. | Setting of data collection | Where was the data collected? e*.g. home, clinic, workplace* **Page 8 and 9** |
| 15. | Presence of non-participants | Was anyone else present besides the participants and researchers? **No** |
| 16. | Description of sample | What are the important characteristics of the sample? *e.g. demographic data, date* **Page 11 and 12** |
| Data collection |  |  |
| 17. | Interview guide | Were questions, prompts, guides provided by the authors? Was it pilot tested? **Supplementary File 3** |
| 18. | Repeat interviews | Were repeat interviews carried out? If yes, how many? **Yes - three** |
| 19. | Audio/visual recording | Did the research use audio or visual recording to collect the data? **Audio recording** |
| 20. | Field notes | Were field notes made during and/or after the interview or focus group? **Page 10** |
| 21. | Duration | What was the duration of the interviews or focus group? **Page 11** |
| 22. | Data saturation | Was data saturation discussed? **Not applicable** |
| 23. | Transcripts returned | Were transcripts returned to participants for comment and/or correction? **Yes – page 10** |
| **Domain 3: analysis and findings**z |  |  |
| Data analysis |  |  |
| 24. | Number of data coders | How many data coders coded the data? **Page 10** |
| 25. | Description of the coding tree | Did authors provide a description of the coding tree? **Page 11** |
| 26. | Derivation of themes | Were themes identified in advance or derived from the data? **Page 10 and 11** |
| 27. | Software | What software, if applicable, was used to manage the data? **Page 10** |
| 28. | Participant checking | Did participants provide feedback on the findings? **Page 10** |
| Reporting |  |  |
| 29. | Quotations presented | Were participant quotations presented to illustrate the themes / findings? Was each quotation identified? e*.g. participant number* **Page 12-19** |
| 30. | Data and findings consistent | Was there consistency between the data presented and the findings? **Page 12-19** |
| 31. | Clarity of major themes | Were major themes clearly presented in the findings? **Page 12-19** |
| 32. | Clarity of minor themes | Is there a description of diverse cases or discussion of minor themes? **Page 12-19** |

***** Allison Tong, Peter Sainsbury, Jonathan Craig, Consolidated criteria for reporting qualitative research (COREQ): a 32-item checklist for interviews and focus groups, International Journal for Quality in Health Care, Volume 19, Issue 6, December 2007, Pages 349–357, <https://doi.org/10.1093/intqhc/mzm042>
