# Supplementary material for: An aboriginal community-controlled health organization model of service delivery: qualitative process evaluation of the Tulku wan Wininn mobile clinic
Source: Int J Equity Health. 2022 Nov 16;21:163. doi: 10.1186/s12939-022-01768-4 (PMC9667861; doi:10.1186/s12939-022-01768-4)
Supplement: Supplementary file 2 — Supplementary Material 2. Application of the CONSIDER Statement. [file 12939_2022_1768_MOESM2_ESM.docx]

**Supplementary File 2 – Application of the CONSIDER Statement^**

| **Domain** | **Process evaluation of the Tulku wan Wininn mobile clinic** |
| --- | --- |
| **Governance** | |
| ***1. Describe partnership agreements between the research institution and Indigenous-governing organisation for the research, (e.g., Informal agreements through to MOU (Memorandum of Understanding) or MOA (Memorandum of Agreement)).*** | Deakin University (DU) and Budja Budja Aboriginal Cooperative (BBAC) do not have a MOU. Rather multiple letters of support were provided by BBAC to submit with ethics and funding applications which reflect the nature of the partnership which includes clinical placements, evaluation work and cultural competency training. |
| ***2. Describe accountability and review mechanisms within the partnership agreement that addresses harm minimisation.*** | Part of working in a respectful partnership means that all work, including drafts of manuscripts, is reviewed by BBAC prior to dissemination. |
| ***3. Specify how the research partnership agreement includes protection of Indigenous intellectual property and knowledge arising from the research, including financial and intellectual benefits generated (e.g., development of traditional medicines for commercial purposes or supporting the Indigenous community to develop commercialisation proposals generated from the research).*** | The project had little scope for commercial benefit. The NHMRC Human Research Ethics Application (HREA) submitted to DUHREC was focused on how cultural knowledge would be managed through the project. |
| **Prioritisation** | |
| ***4. Explain how the research aims emerged from priorities identified by either Indigenous stakeholders, governing bodies, funders, non-government organisation(s), stakeholders, consumers, and empirical evidence.*** | The evaluation was designed in conjunction with BBAC leaders in the context of needing to understand the acceptability of the mobile clinic as a model of service delivery. This is part of a larger body of work led by BBAC in partnership with Deakin Rural Health (DU) around evaluating different ACCHO service models to improve health care accessibility for Aboriginal and Torres Strait Islander community members in the region. |
| **Relationships (Indigenous stakeholders/participants and Research team)** | |
| ***5. Specify measures that adhere and honour Indigenous ethical guidelines, processes, and approvals for all relevant Indigenous stakeholders, recognising that multiple Indigenous partners may be involved, e.g., Indigenous ethics committee approval, regional/national ethics approval processes.*** | As there is no formal process for having research projects involving Aboriginal and/or Torres Strait Islander Peoples reviewed by an Indigenous-specific ethics committee in Victoria, local processes were used to ensure the cultural appropriateness and safety of the research project. This involved having BBAC review all documents prior to submitting a formal ethics application to DUHREC and discussing other ethical issues at length. |
| ***6. Report how Indigenous stakeholders were involved in the research processes (i.e., research design, funding, implementation, analysis, dissemination/recruitment).*** | Evaluation design was agreed upon by BBAC and DRH, with input from Aboriginal academics from DU. The funding submission had input from BBAC and DU researchers, including Aboriginal academics. |
| ***7. Describe the expertise of the research team in Indigenous health and research.*** | The DU research team included public health and clinical experience in Aboriginal and Torres Strait Islander health. This included a participatory action research project which was undertaken in collaboration with another rural ACCHO to engage the perspectives of Aboriginal and/or Torres Strait Islander community members around chronic disease. |
| **Methodologies** | |
| ***8. Describe the methodological approach of the research including a rationale of methods used and implication for Indigenous stakeholders, e.g., privacy and confidentiality (individual and collective).*** | A community-based qualitative process evaluation methodology guided by the CONSIDER statement was used. This involved having BBAC personnel involved in the study design, interviews, and recruitment of Aboriginal and Torres Strait Islander clients to the interviews. Due to the overlap of BBAC personnel in the study components, strict ethical data management protocols were adhered to. This included storing de-identified interview transcripts on a DU password protected computer drive and only sharing this with the respective participants. Data analysis and interpretation were also only shared with BBAC personnel in the form of summaries and an evaluation report in order to protect the confidentiality of all participants. |
| ***9. Describe how the research methodology incorporated consideration of the physical, social, economic and cultural environment of the participants and prospective participants. (e.g., impacts of colonisation, racism, and social justice). As well as Indigenous worldviews.*** | A community-based process evaluation methodology guided by the CONSIDER statement ensured all aspects of the study were overseen by BBAC personnel. Further, the leadership of Aboriginal academics in the study ensured the study was conducted in a manner which was culturally appropriate. In the context of ongoing COVID-19 lockdowns and the inability to meet face-to-face with Aboriginal and/or Torres Strait Islander community members, this involved having BBAC health service personnel invite clients to the study, meeting via telephone, and sometimes having multiple telephone interviews with clients to develop rapport. |
| **Participation** | |
| ***10. Specify how individual and collective consent was sought to conduct future analysis on collected samples and data (e.g., additional secondary analyses; third-parties accessing samples (genetic, tissue, blood) for further analyses).*** | Prior to each interview, consent forms with plain language statements were provided to participants. The content of these were also agreed upon by BBAC and DU Aboriginal academics. |
| ***11. Describe how the resource demands (current and future) placed on Indigenous participants and communities involved in the research were identified and agreed upon including any resourcing for participation, knowledge, and expertise.*** | As the evaluation received funding, resourcing demands were considered and accounted for. This included funding for reimbursement for Aboriginal and/or Torres Strait Islander clients participating in the interviews. |
| ***12. Specify how biological tissue and other samples including data were stored, explaining the processes of removal from traditional lands, if done, and of disposal.*** | Not applicable to this study. |
| **Capacity** | |
| ***13. Explain how the research supported the development and maintenance of Indigenous research capacity (e.g., specific funding of Indigenous researchers).*** | Not applicable to this study. |
| ***14. Discuss how the research team undertook professional development opportunities to develop the capacity to partner with Indigenous stakeholders?*** | This process took place through regular meetings between BBAC and DRH during the evaluation timeline and following. Non-Indigenous researchers also participated in cultural awareness training hosted by BBAC to develop skills and an understanding of the local community. |
| **Analysis and interpretation** | |
| ***15. Specify how the research analysis and reporting supported critical inquiry and a strength-based approach that was inclusive of Indigenous values.*** | Research analysis and reporting were supported by an Aboriginal Associate Research Fellow which guided interpretation of findings within a strengths-based approach. |
| **Dissemination** | |
| ***16. Describe the dissemination of the research findings to relevant Indigenous governing bodies and peoples.*** | A one-page summary of the evaluation was provided to clients who participated in the interviews in order to inform them of how their feedback was understood. An internal evaluation report with all findings was provided to BBAC. |
| ***17. Discuss the process for knowledge translation and implementation to support Indigenous advancement (e.g., research capacity, policy, investment).*** | Findings from the evaluation were provided to BBAC in the form of a report and to clients in the form of a one-page summary. |

**^** Huria T, Palmer SC, Pitama S, Beckert L, Lacey C, Ewen S, Smith LT. Consolidated criteria for strengthening reporting of health research involving indigenous peoples: the CONSIDER statement. *BMC medical research methodology*. 2019;19(1):173.
