# Supplementary material for: An aboriginal community-controlled health organization model of service delivery: qualitative process evaluation of the Tulku wan Wininn mobile clinic
Source: Int J Equity Health. 2022 Nov 16;21:163. doi: 10.1186/s12939-022-01768-4 (PMC9667861; doi:10.1186/s12939-022-01768-4)
Supplement: Supplementary file 3 — Supplementary Material 3. Interview Guides. [file 12939_2022_1768_MOESM3_ESM.docx]

**Supplementary File 3 – Interview Guides**

**Interview guide for health service personnel**

***Topic: Contextual factors***

- - - 1. Tell me about your role and how you have been involved in the implementation of the Tulku Wan Wininn mobile clinic

*Prompts – What clinical services do you provide? How long have you been working with Budja Budja Aboriginal Cooperative?*

- - - 1. What is your understanding of the circumstances which initiated the need for a mobile clinic van?

*Prompts – What was your involvement in the conceptualization of a mobile clinic van? How involved were Community members in discussions around the need for a mobile clinic van?*

- - - 1. How would you describe the initial implementation of the mobile clinic?

*Prompts – What are some contextual barriers and enablers to implementation? What cultural factors have affected implementation? Were there any teething issues?*

***Topic: Implementation***

- - - 1. What changes to the mobile clinic service plan have been made since implementation?

*Prompts – Why were these changes made? How have these changes impacted service delivery? Do you foresee any further changes being made?*

***Topic: Adoption***

- - - 1. How engaged do you think Community members are with the mobile clinic?

*Prompts – Do you have any specific examples you could share with me? How could other Community members be engaged in accessing services? Why do you think Community members may not be accessing the mobile clinic services? How could engagement issues be overcome?*

- - - 1. What are your observations regarding Community acceptability of the mobile clinic as a model of health care?

*Prompts – Do you have any specific examples you could share with me? Have any Community members expressed dissatisfaction with the mobile clinic? Are there any changes which need to be made?*

***Topic: Maintenance***

- - - 1. What is required for the ongoing implementation of the mobile clinic?

*Prompts – How important are partnerships between health services and universities? How important is funding, local support and engagement or other factors?*

- - - 1. What challenges do you foresee for the ongoing implementation and sustainability of the mobile clinic?

*Prompts – How could these challenges be addressed?*

**Any other comments or thoughts to share?**

**Interview guide for clients**

- Introduce researcher and purpose of evaluation
- Discuss PLS and consent form

If community member is happy to proceed, then ask following questions. Also provide community member with the option to have the interview at another time which is more convenient.

1. **Could you please tell us about your experience of visiting the Tulku wan Wininn mobile clinic?**

(Optional prompts – how important is having health care available nearby, to you? Do you feel comfortable attending the mobile clinic?)

1. **What changes do you think need to be made to the Tulku Wan Wininn mobile clinic that would help you to look after your health?**

(Optional prompts – what do you like about the service? What do you not like?)

**Option to proceed to question 3.**

1. **How do you think the Tulku wan Wininn mobile clinic is being received by your community?**

(Optional prompts- would you recommend the mobile clinic to friends and family?)

***Is there anything else you would like to share?***

Thank you for your time. We look forward to meeting with you in person once COVID-19 lockdown has been lifted.
